# The weak magnetic field inhibits the supramolecular self-ordering of chiral molecules

**DOI:** 10.1038/s41598-020-74297-1

**Published:** 2020-10-13

**Authors:** Sergey V. Stovbun, Anatoly M. Zanin, Aleksey A. Skoblin, Dmitry V. Zlenko

**Affiliations:** 1 N.N. Semenov Federal Research Center for Chemical Physics RAS, Moscow, Russia; 2 Faculty of Biology, M.V. Lomonosov Moscow State University, Moscow, Russia

**Keywords:** Biophysics, Supramolecular assembly

## Abstract

The magnetic field can affect processes in the non-magnetic systems, including the biochemical reactions in the living cells. This phenomenon becomes possible due to the fermionic nature of an electron and significant energy gain provided by the exchange interactions. Here we report the inhibition effect of the magnetic field on the processes of the chiral supramolecular, i.e., macroscopic self-ordering in the non-magnetic model system. The observed effect is in tune with the reports on the influence of the magnetic field on the adsorption of the chiral molecules, which was explained by the effect of the chirally-induced spin-selectivity and the inhibition of the chemical reactions caused by the singlet-triplet conversion. The magneto sensitivity of the process of the chiral self-ordering directly indicates its spin-polarization nature. Tacking together all of the results in the field, we can propose that the chirality-driven exchange interactions guide the selection of the chiral molecules and explain their prevalence in the living matter. It is also probable that these forces have played a critical role in the origin of life on Earth.

The interaction of the magnetic field with the spin of the electrons and their spatial distribution leads to the well known and rather weak para- and diamagnetic effects. Besides that, there is another much more significant effect related to the fermionic nature of the electrons, which means that the permutation of two electrons changes the sign of the system’s wave function. In the two-electron system, the wave function could be represented as a product of the spin and spatial parts, and one of them is symmetrical (even) while other—antisymmetrical (odd) concerning the particles’ permutation. The even spin-state has the total spin of unity and is known as the triplet state, while the odd one has the zero total spin and known as the singlet state. The interaction with the external magnetic field may cause the singlet-triplet conversion, which means the change of the parity of the spin part of the wave function. So, the spatial part also must change its parity from even to odd. The odd spatial wave function vanishes when the coordinates of the electrons coincide, so, in the triplet state, the electrons stay far from each other, and the energy of their electrostatic repulsion reduces as compared to the singlet state, which reduces the full energy of the system. This effect is well known as the change in the exchange interaction between the electrons^1^. The change in the energy of the exchange interactions can be significant, and reach up to

1 $$\begin{aligned} e^2/(4\pi \varepsilon _0r) \approx 1\,{\mathrm{eV}}~\sim 40\,{\mathrm{kT}}, \end{aligned}$$

where *e* is the charge of an electron, $\varepsilon _0$ is the vacuum permittivity, *k* is the Boltzmann constant, *T* is the temperature equal to 300 K, and *r* is the characteristic distance of 1 nm. This particular effect is responsible for the ferro-, ferri-, and anti-ferromagnetizm.

For the last 50 years, the was published a lot of the reports on the interaction of the “nonmagnetic” materials with the magnetic field. First of all, the aromatic parts of the molecules exhibit pronounced diamagnetic properties and can orient in the magnetic field^2^. Besides that, the surfaces of the lipid membranes tend to align perpendicular to the very small magnetic field insufficient for the manifestation of the anisotropy of the magnetic susceptibility^3,4^ that reflects their unusual sensitivity to the magnetic field. Such effects were observed for outer segments of retinal rods^5^, chloroplasts^6^ and bacterial chromatophores^7^, purple membranes^8^, and even for the black lipid membranes^9^.

The sensitivity of the non-magnetic systems to the magnetic field could be provided by the exchange interactions in the spin-polarized systems^3–9^. The spin polarization accompanies the charge polarization when a molecule interacts with another molecule or with a surface^10,11^. In the elongated, helical molecules exposed to the external electric field parallel to their axis, the spin polarization appears as a result of the chirally-induced spin selectivity (CISS) effect. The latter is caused by the strong correlation between the spin of the electron and the anisotropy of its mobility along the axis of the elongated helical molecule^11,12^. CISS manifests itself in various processes, such as the charge transfer^13–16^, Red/Ox reactions^17^, and biorecognision^10,18^. For our knowledge, CISS was never observed for achiral molecules.

The spin polarization appears to be crucial for biradical reactions, among other ones including photochemical conversions in the condensed phase^19–23^. The nuclear spin can also affect the rate of the chemical reactions, which was described as a magnetic isotope effect^24,25^. The nuclear spin provides for its own heterogenous magnetic field which can interact with other molecular magnetic fields and alter the structure of the energy levels. Therefore, the interaction of the spin of the paramagnetic nucleus with the spins of the unpaired electrons can promote the singlet-triplet conversion that can even inhibit the reaction, as it was shown for $^{13}$C dibenzyl ketone^26^. The same mechanism provides for the inhibition of the nucleic acid synthesis by paramagnetic ions, such as $^{25}$Mg, $^{43}$Ca, and $^{67}$Zn^27^.

The magnetic field can affect the interaction of the helical molecules with the substrate^28,29^. The self-assembled monolayers of the chiral molecules on the surface of ferromagnetic material appeared to be able of switching of the magnetization of the ferromagnetic substrate. This effect was ascribed to the short-range coupling of the wavefunctions of the ferromagnetic and chiral monolayer, and the spin selectivity was provided by the chirality and helicity of the adsorbed molecules^28^. The same effect could be turned upside down, and provide for the enantiospecific adsorption on the magnetized surfaces^29^ and, probably, even the segregation of enantiomers. The latter was in part demonstrated for small and chiral, but not helical molecule of the amino acid cysteine^29^. The problem of the enantiomers segregation is one of the crucial problems of the Life development on Earth^30–32^, and the magnetic field driven segregation can be one of the possible solutions. Therefore, the influence of the magnetic field on the processes of the self-organizing in chiral systems has some additional fundamental sense.

Over the last years, we investigated the processes of the self-ordering and structures’ formation in the cooling or evaporating solutions of the trifluoroacetylated $\alpha$-aminoalcohols (TFAAAs). The molecules of TFAAAs are chiral, low-molecular weight (Fig. 2), and have more or less isometrical shape (Fig. 3). TFAAAs form thin ($\sim$ ${\upmu }\hbox {m}$) and highly elongated (up to millimeters) helical supramolecular structures called “strings”^33–35^. The ultra-thin ($\sim 1$ nm) elementary strings twist together to form several heirarchical levels of thicker strings of a diameter up to several microns, and in particular cases—up to 100 ${\upmu }\hbox {m}$^32^. The supramolecular, heirarchical sytucture of the strings resembles the structure of boilogical macromolecules and their aggregatesTverdislov2017, Stovbun2018StrPhen. However, there was no data on the sensitivity of the strings’ formation to the external magnetic field, while the effects of the spin polarization^10,11,18^ can affect the systems composed of the non-magnetic at the first glance molecules. Moreover, both the strings’ formation^33^ and the CISS-based^11^ effects are observed only in chirally pure systems, or systems demonstrating spontaneous resolution of enantiomers^35^.

**Figure 1:**
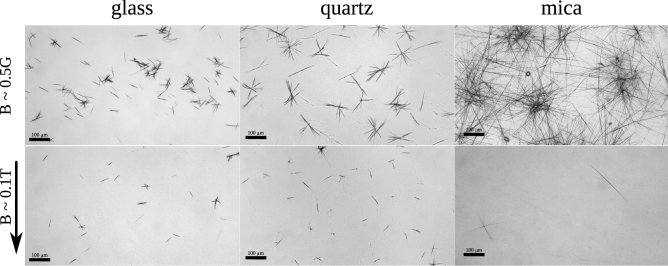
The optical microscope images of the TFAAA strings on the surface of the glass, quartz and mica. Strings were growing with (bottom row) or without (top row) the external magnetic field with the induction $B = 0.18$ T parallel to the glass surface (direction indicated by the arrow) at $20\,^\circ$C.

The main goal of the presented work was to verify if the weak magnetic field is capable of influencing the supramolecular and, so, a macroscopic self-ordering process in the soft matter. We have investigated the formation of the strings in xerogels formed in the course of the evaporation of the heptane solution having the concentration of 0.6 mg/ml on the surface of the glass, quartz, and mica. The TFAAA-7 molecule was chosen, as it contains the aromatic phenyl group (Fig. 2) that should enhance its diamegnetic properties and magnetic sensitivity of the system^2^. Trying to avoid the effect of the magnetic field on the selective adsorption of the chiral molecules on the substrate^28,29^, we used the magnetic field parallel to the surface of the substrate. The magnetic field induction (*B*) was rather low ($B \approx 0.18$ T. The magnitude of 0.18 T was chosen following several reasons:

1. The energy of the interaction of the electron with such a field is much smaller than the thermal energy (*kT*) and could be estimated as:
  2 $$\begin{aligned} \mu _B B \approx 1.0 \cdot 10^{-5}\,{\mathrm{eV}} \approx 4 \times 10^{-4} kT, \end{aligned}$$
  where $\mu _B \approx 5.8 \cdot 10^{-5}$ eV/T—the Bohr magneton;
2. The value of 0.18 T is comparable to the intermolecular magnetic fields^37^ and can affect the chemical reaction^27^;
3. The magnetic field of $D \sim 0.2$ T is a threshold for the orientation effects in lipid bilayers^9^.

The examination of the xerogels obtained in the external magnetic field demonstrated that even a relatively weak magnetic field of 0.18 T effectively inhibited the formation of the strings on the substrate. This effect was independent of the material of the substrate, and the direction of the magnetic field (parallel to the surface of the substrate), while the distribution of the directions of the growth of the string was isotropic (Fig. 1). For each type of the substrate, we have made 50 independent experiments in the presence of the magnetic field and the same quantity of the control ones. The amount of the strings was counted in the center of each specimen and the average net amount of the strings was summarized in Table 1.

**Table 1:** The average specific surface concentration of strings ($\times 10^{-7}$, $\hbox {m}^{-2}$) on the different substrate in the presence or absence of the external magnetic field ($B = 0.18$ T).

	Glass	Quartz	Mica
$B \sim 0.5$ G	$35.2 \pm 17.1$	$33.4 \pm 15.6$	$78.3 \pm 41.2$
$B \sim 0.2$ T	$6.2 \pm 3.8$	$8.5 \pm 5.2$	$0.8 \pm 0.5$

The standard deviations were used as the error of mean.

There are a lot of experimental results indicating the role of the exchange interactions in the behavior of the biological systems and their susceptibility to the external magnetic fields:

1. Lipid bilayers tend to align straight across the magnetic field that was interpreted as an effect of the spin polarization and exchange interactions^3–9^;
2. The effect of the chirally-induced spin selectivity was directly observed in the biopolymers^10,11^;
3. The magnetic field effectively inhibits the growth of the supramolecular strings in the solutions of TFAAA-7. Given that the cohesion energy of the monomer in the string is at least 20 kJ/mole (i.e., $\sim 8$ kT)^33^, while the energy of the interaction of the external field applied with the electrons was only about $10^{-4}$ kT, direct interaction with the magnetic field cannot provide for the effect observed.
4. The exchange interaction seems to be pivotal for the processes of the bio-recognition^9,18^ and bio-discrimination^38^, which are essential for the modern life and its development^30,39^. At the same time, current physical models do not correctly describe the enantioselectivity and binding energies in biorecognition^40,41^. The bio-discrimination of the enantiomers is also hard to explain based on the simple van der Waals interactions, as it requires the energy gain of about 10–15 kT^38,42^. However, these difficulties could be in part compensated by accounting for the enantioselective interactions^10^.
5. The quasi-one-dimensional supramolecular strings spontaneously formed by TFAAS have a helical structure and organize into a complex hierarchy of the structural levels sequentially changing the sign of chirality^33^. Such an organization is peculiar for the biological macromolecules^36,43^ and seems to be forced by the chirality itself. Moreover, formation of the strings by TFAAAs can be accompanied by the spontaneous resolution of the enatiomers^44^. Similar helical structures were found in the solutions of carbohydrates^31^ and aminoacids^45^.
  Therefore, based on the observations listed above, we can put forward the hypothesis that the chirally-induced spin selectivity and the related effects driven by the exchange interactions could have been playing a significant role in the germination and development of Life on Earth^31,32^.

## Methods

### Samples preparation

**Figure 2:**
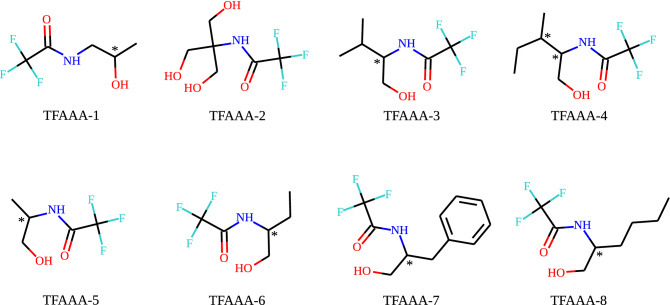
The structural formulas of the trifluoroacetylated $\alpha$-aminoaclohols that are able to form strings (except of the achiral TFAAA-2). In this work, we used TFAAA-7 having the phenyl radical.

The range of the chiral TFAAAs (Fig. 2) were synthesized, as was described previously^46^. The dry samples of the TFAAA-7 (0.6 mg/ml) were dissolved in heptane (ChimMed, Russia) heated to 60–$70\,^\circ$C under intensive stirring. We observed the decrease of the number of strings after several dozens of the heating-cooling cycles, so, here we used only the samples of the TFAAAs dissolved and heated for the first time. 15 ${\upmu }\hbox {l}$ of the hot solution was dropped on the surface of the glass, quartz, or mica that was preliminarily thermostated at the temperature of $20\,^\circ$C. Then, the samples were placed back in the thermostat either between the magnets or without them. The field was directed parallel to the surface of the substrate, but the angle in respect to the borders of the specimen was varied in the range $\pm 60\,^\circ$C. After heptane evaporation, the center of the drop was analyzed under the optical microscope (MIKMED-6, LOMO, Russia) under relatively low magnification ($\times 100$–$\times 400$).

The external magnetic field was induced by two cylindrical Nd-magnets (55 mm in diameter and 25 mm in thick, N38 alloy), placed concentrically at the distance of 37 mm between the surfaces. The sample was placed on the axis of the magnets, in the middle between them. The magnetic field induction was approximately equal to $0.18$ T.

**Figure 3:**
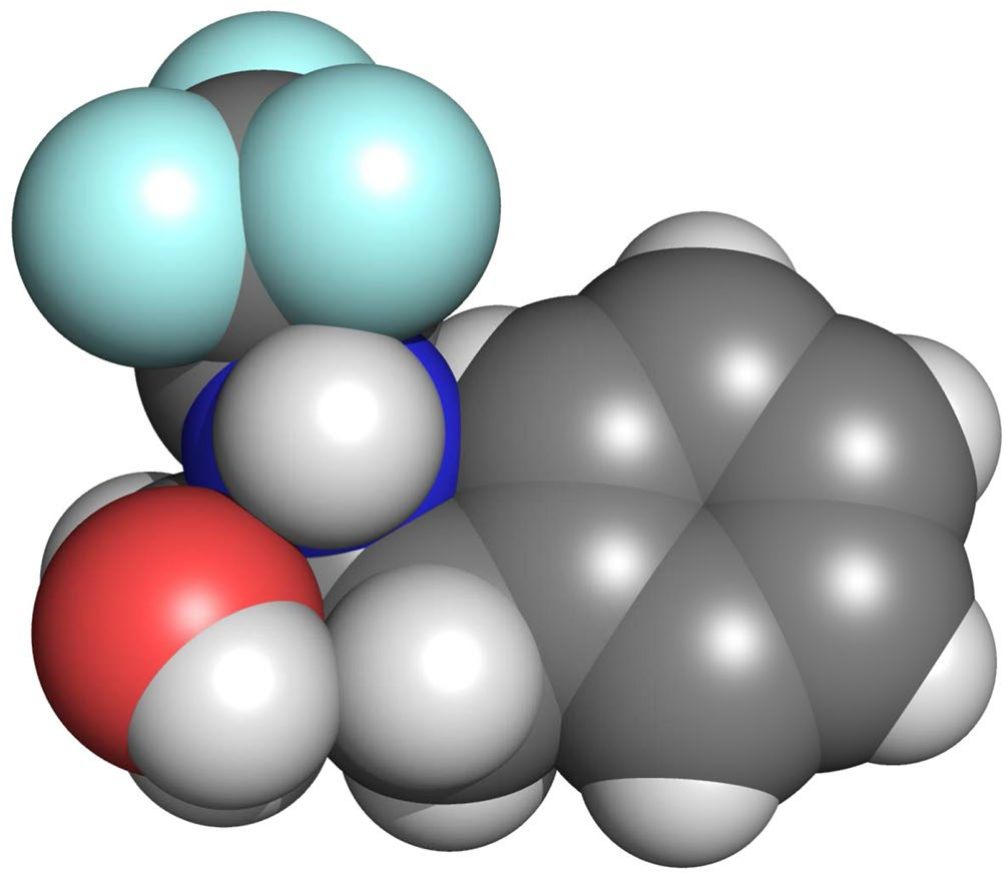
Van der Waals representation of the TFAAA-7 molecule. Red color represents the oxygen atoms, dark-blue—nitrogen, light-blue—fluorine, gray—carbon, and white—hydrogen.

### Measurement of the magnetic field induction

To verify the real magnetic field intensity we have used a linear-output Hall sensor (AD22151, Analog Devices, USA) coupled with 24-bit ADC (AD7799, Analog Devices, USA). STM32F103 microcontroller (STMicroelectronics, Switzerland) was used for the ADC operation and communication with a PC (all of the raw files are available at GitHub: github.com/dvzlenko). According to the specification, AD22151 Hall sensor has the offset error of 0.6 mT, while we registered the offset error of $\sim 0.35$ mT.

The magnetic field induction measured exactly on the axis of the magnet was $181.8 \pm 0.7$ mT. Taking into account the diameter of the drop of the heptane solution on the substrate (8–10 mm), the real induction on the edges of the drop would be about 188 mT. However, all of the images provided (Fig. 1) were taken from the center of the specimen and corresponds to $B \approx 0.18$ T.
